# Deep learning-based label-free imaging of lymphatics and aqueous veins in the eye using optical coherence tomography

**DOI:** 10.1038/s41598-024-56273-1

**Published:** 2024-03-13

**Authors:** Peijun Gong, Xiaolan Tang, Junying Chen, Haijun You, Yuxing Wang, Paula K. Yu, Dao-Yi Yu, Barry Cense

**Affiliations:** 1 Key Laboratory for Biomedical Engineering of Ministry of Education, Zhejiang University, Hangzhou, 310027 China; 2 School of Software Engineering, South China University of Technology, Guangzhou, 510006 China; 3 Department of Electrical, Electronic and Computer Engineering, School of Engineering, The University of Western Australia, Perth, WA 6009 Australia; 4 Key Laboratory of Big Data and Intelligent Robot (SCUT), Ministry of Education, Guangzhou, 510006 China; 5 Centre for Ophthalmology and Visual Science, The University of Western Australia, Perth, WA 6009 Australia; 6 Lions Eye Institute, Nedlands, WA 6009 Australia; 7 Department of Mechanical Engineering, Yonsei University, Seoul, 03722 Republic of Korea

**Keywords:** Imaging and sensing, Optical imaging

## Abstract

We demonstrate an adaptation of deep learning for label-free imaging of the micro-scale lymphatic vessels and aqueous veins in the eye using optical coherence tomography (OCT). The proposed deep learning-based OCT lymphangiography (DL-OCTL) method was trained, validated and tested, using OCT scans (23 volumetric scans comprising 19,736 B-scans) from 11 fresh ex vivo porcine eyes with the corresponding vessel labels generated by a conventional OCT lymphangiography (OCTL) method based on thresholding with attenuation compensation. Compared to conventional OCTL, the DL-OCTL method demonstrates comparable results for imaging lymphatics and aqueous veins in the eye, with an Intersection over Union value of 0.79 ± 0.071 (mean ± standard deviation). In addition, DL-OCTL mitigates the imaging artifacts in conventional OCTL where the OCT signal modelling was corrupted by the tissue heterogeneity, provides ~ 10 times faster processing based on a rough comparison and does not require OCT-related knowledge for correct implementation as in conventional OCTL. With these favorable features, DL-OCTL promises to improve the practicality of OCTL for label-free imaging of lymphatics and aqueous veins for preclinical and clinical imaging applications.

## Introduction

The lymphatic system provides important biological functions in humans, including supporting immune response, fatty acid absorption, and drainage of interstitial fluid. The drainage function in particular enables the conjunctival lymphatics in the eye to play an important role in the bleb-forming surgical treatment of glaucoma^1^, which creates a drainage pathway to release the abnormally high eye pressure due to the excessive aqueous humor (AH) in the eye^2^, leading to a filtering bleb in the subconjunctival tissue^3^. Although the subsequent drainage from the bleb is not fully understood, the integrity and density of conjunctival lymphatics are considered critical for the longevity of the created drainage pathway and good treatment outcomes^3^. Imaging can help to advance our knowledge of the role of the conjunctival lymphatics in glaucoma surgery and to optimize the selection of regions with healthy lymphatics for surgery that facilitate drainage.

Progresses in methods for imaging lymphatics have led to techniques based on near-infrared fluorescence imaging (indocyanine green lymphangiography), ultrasound imaging, computed tomography, magnetic resonance imaging and positron emission tomography^4–7^. Whilst they are capable of imaging large tissue areas/volumes in vivo, they can only visualize the large lymphatics (e.g., lymph nodes and large collecting vessels) due to their limited resolutions. To image the micro-scale lymphatics (e.g., small collecting vessels and capillaries), higher imaging resolutions can be provided using optical contrast, such as photoacoustic imaging which uses optical illumination and ultrasonic detection^4,8^. Optical microscopy can achieve even higher resolutions, but it typically requires histological tissue processing that suits mainly ex vivo imaging applications. Despite these advances, label-free imaging of the lymphatics is yet difficult to achieve as the current methods largely require exogenous contrast agents to label the lymphatics for imaging.

Using the intrinsic optical transparency (i.e., low scattering) of lymph, optical coherence tomography lymphangiography (OCTL) is emerging as a label-free method for imaging lymphatics with micrometer-scale resolutions^6,9^. The transparency leads to a markedly low optical coherence tomography (OCT) backscattering signal, which creates the vessel contrast relative to the surrounding tissue with higher OCT signals^9^. OCTL uses image segmentation to identify low-signal vessel structures in OCT scans, leading to several variants based on thresholding or morphological image processing. Thresholding can be performed by applying a constant threshold to the whole OCT volumetric scan^10^, but it is subject to artifacts especially for deep tissue with significantly attenuated signal^11–13^. Such artifacts have been mitigated by adaptive thresholding^14^, which estimates adaptive thresholds in local depth windows with the window size determined by assuming the vessel sizes. Alternatively, compensation of the depth attenuation enables the use of a constant threshold to the whole OCT volume to reduce attenuation-induced artifacts^15,16^. Morphological image processing has been implemented with Hessian filtering^17^, which uses the high-order shape information to identify long, thin structures as vessels. These OCTL variants have been demonstrated on skin and eye tissue in vivo and ex vivo^16,18^.

An additional application of OCTL is to image the aqueous vein vessels in the sclera of the eye^19,20^. These aqueous vein vessels form a critical part of the outflow pathway of the transparent AH in the eye, which regulates the intraocular pressure (IOP) by the balance between the production and outflow of AH^21^. When the AH outflow resistance is increased, IOP is elevated and becomes a major risk factor in glaucoma. Imaging the AH outflow pathway therefore bears great importance for investigating the pathogenesis and potential therapeutic strategy of glaucoma^2^. The aqueous vein vessels in the sclera provide an important window to image the AH outflow pathway as they are approximately within the imaging depth of OCT (up to ~ 1 mm) and present a similar optical transparency to the lymphatics^19,20^, enabling the use of OCTL for imaging.

Overall, although the imaging potential has been shown for lymphatics and aqueous veins, OCTL still presents limitations associated with attenuation-induced artifacts, the complex and time-consuming OCT signal modelling for attenuation compensation, or the morphological segmentation assumptions of the vessel shapes. Consequently, current OCTL methods are not yet practical enough to be directly used by clinicians, impeding preclinical and clinical imaging applications.

Deep learning, a class of machine learning algorithms to progressively extract high-level features using multiple layers, has been extensively used for image segmentation^22^. Among various deep learning neural networks, convolutional neural networks (CNNs) have been found highly effective for medical image analysis. The hidden layers between the input and output layer in CNNs, comprise multiple convolutional layers interspersed with pooling layers, which are followed by a normal multilayer neural network (i.e., fully connected layers). This characteristic architecture can effectively preserve the structural information in the neighboring pixels (or voxels) of the target pixel (voxel), which is important for medical image segmentation^22^. Methods based on CNNs have been applied to the segmentation of structures ranging from large organs to small cells^22–24^. They also hold promise for segmentation of lymphatics/aqueous veins in OCT scans to improve the practicality of OCTL, but have not been explored so far. A recent study by Lai et al*.* incorporated deep learning as a supplementary step into their Hessian filtering-based OCTL^25^. However, deep learning was only used to segment and remove a single layer of cartilage with confounding low OCT signal in the mouse ear skin and not employed for direct segmentation of the more complex lymphatic vessels.

In this paper, we present the first adaptation of deep learning for OCTL imaging based on the U-Net network. U-Net, a variant of CNNs^26^, was selected to develop a deep learning-based OCTL (DL-OCTL) method as U-Net improves the utilization rate of the training data and carries out end-to-end training in the case of a small number of samples to improve the segmentation accuracy. OCT scans (23 volumetric scans comprising 19,736 B-scans) from 11 fresh ex vivo porcine eyes were used for the training, validation and testing of the DL-OCTL method, for imaging the lymphatic and the aqueous vein vessels in the eye. The segmentation results by DL-OCTL for the testing group were compared to a previously reported conventional OCTL method based on thresholding with attenuation compensation that provided the vessel labels^15^, showing very comparable vessel segmentation with reduced imaging artifacts that were caused by inaccurate OCT signal modelling associated with tissue heterogeneity, accelerated processing speed and streamlined processing.

## Methods

### OCT scanning

A commercial spectral-domain OCT system (an upgraded TELESTO II, Thorlabs Inc., Newton, USA) was used for tissue scanning with a center wavelength of 1300 nm^27^. The system provided an axial and lateral imaging resolution of, respectively, 5.5 µm in air (i.e., 3.9 µm in tissue assuming a refractive index of ~ 1.4 at 1300 nm) and 13 µm in air and tissue (using the LSM03 scan lens from Thorlabs Inc.) as defined by the vendor. An A-scan (depth scan) rate of 48 kHz or 76 kHz was used to collect 3-D OCT scans non-invasively with non-contact imaging so as to avoid pressure on the eye samples which could collapse the vessels. The imaging field-of-view (FOV) for a 3-D scan was either 3 × 3 mm or 6 × 4.5 mm in the lateral (*x*–*y*) directions, depending on the local tissue surface geometry. The number of sampled A-scans within the FOV was either 1024 × 1024 or 1024 × 600 along *x* and *y* directions. The total imaging depth (*z* direction) range was 3.6 mm in optical path length (i.e., ~ 2.6 mm in physical length assuming a refractive index of ~ 1.4).

**Figure 1:**
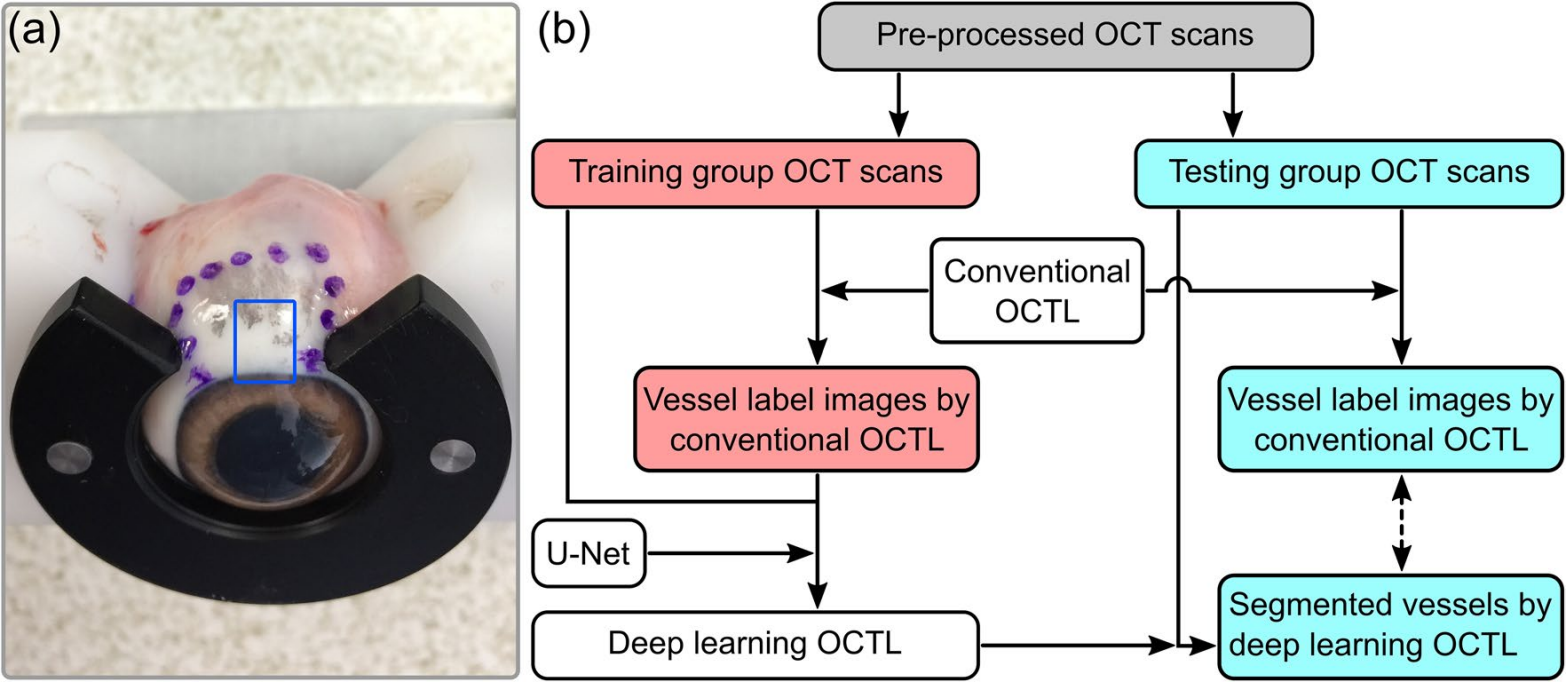
(**a**) Experimental setup with the tissue region for scanning indicated by the blue outline. (**b**) Summary of the data processing flow. Pre-processed OCT scans were divided into the training and testing groups. Training group was processed by conventional OCTL to generate the vessel labels for developing the DL-OCTL method based on U-Net. DL-OCTL was then applied to the testing group with the results compared to those from conventional OCTL.

Porcine eyes were chosen for ex vivo imaging due to their morphological similarities to human eyes. The eye samples were prepared for OCT scanning using a previous protocol^16^, with approval by the Animal Ethics Committee of The University of Western Australia. In brief, freshly enucleated porcine eyes were obtained from the local abattoir and transported in ice-cold carbogen-bubbled Ringer’s solution in gas-tight containers to the laboratory within an hour of enucleation. A custom-made eye holder was used to allow easy adjustment of the orientation of the eye for imaging of the conjunctiva and sclera, as shown in Fig. 1a. During the sample setup and imaging, care was taken not to touch the selected tissue regions for imaging in order to avoid pressure on the tissue, which could collapse the vessels. Saline drops were applied to hydrate the eye tissue. OCT imaging was completed within ~ 3–4 h of enucleation. In total, 11 porcine eye samples were scanned, including several samples used in a previous study^16^. This led to a total of 23 3-D OCT scans (comprising 19,736 B-scans) collected for OCTL imaging.

### OCT data pre-processing

The recorded raw OCT spectrum data were first processed to construct the OCT scans (i.e., depth-dependent intensity signal based on tissue backscattering) in MATLAB (R2016a, The MathWorks, Inc., Natick, USA). The key processing steps included linear resampling of the spectrum into wavenumber space, subtraction of the background spectrum, spectrum apodization and inverse Fourier transformation. This common processing flow for spectral-domain OCT led to the 3-D complex OCT signal and the logarithm of its magnitude was further calculated. The noise intensity was estimated in the logarithmic signal from a deep region (in *z*) where the OCT signal was fully attenuated (i.e., with only noise). The noise intensity was then subtracted from the logarithmic OCT scan to set the noise level to 0 dB. These pre-processed 3-D logarithmic OCT scans were subsequently used for OCTL processing by the conventional OCTL and DL-OCTL methods.

### Conventional OCTL

To generate the vessel label images for training, validation and testing of the DL-OCTL method, a previous OCTL method based on thresholding with attenuation compensation was first applied to all OCT scans^15^, as shown in the data processing flow in Fig. 1b. This method was extended from OCT-based attenuation imaging that quantifies the rate of the depth decay of the OCT signal to measure the attenuation coefficient^13,28^. In brief, this OCTL method first estimated the attenuation coefficient for each A-scan by fitting the depth-dependent OCT signal to the single-scattering model^11^. Prior to fitting, the system factors, including the confocal function and the sensitivity roll-off function, were first corrected using the calibration scan of a homogeneous, low-scattering phantom^12,29^. Model fitting of the corrected OCT signal was performed using linear least-squares regression to estimate the attenuation coefficient, which was then used to compensate for the attenuation in the corrected OCT signal^15^. The correction, fitting and compensation was performed to each A-scan in the 3-D OCT scan. This led to a 3-D attenuation-compensated OCT scan, in which the lymphatic and aqueous vein vessels maintain their markedly low signal and the surrounding tissue has a nearly constant, high signal. An empirically chosen constant threshold was then applied to the 3-D compensated scan to segment the vessels as a 3-D binary OCTL scan, which provides the vessel labels.

The tissue surface in the original pre-processed OCT scans was detected using a Canny edge detector^30^, and used to align each A-scan in both the pre-processed OCT and the corresponding OCTL scans along depth so that the tissue surface was flattened. B-scans in the flattened 3-D scans were then cropped from the tissue surface to a depth of ~ 500 µm, where the majority of clearly visible vessels were located. In addition, the OCT signal deeper inside the tissue was generally significantly attenuated, which led to a low OCT signal and made it difficult to reliably image vessels, and thus was not used. The binary OCTL scans (i.e., the vessel labels) provided by conventional OCTL and their corresponding original pre-processed OCT scans after surface alignment and cropping were then used for the training, validation and testing of the DL-OCTL method. For visualization, the projection of the vessels was taken along the depth direction in the aligned OCTL scan to form a two-dimensional en face image of the vessel network. All data processing for conventional OCTL was implemented in MATLAB (R2016a, The MathWorks, Inc., Natick, USA) using an Intel® Core™ i9-7900X processor with 32-GB memory.

### DL-OCTL

**Figure 2:**
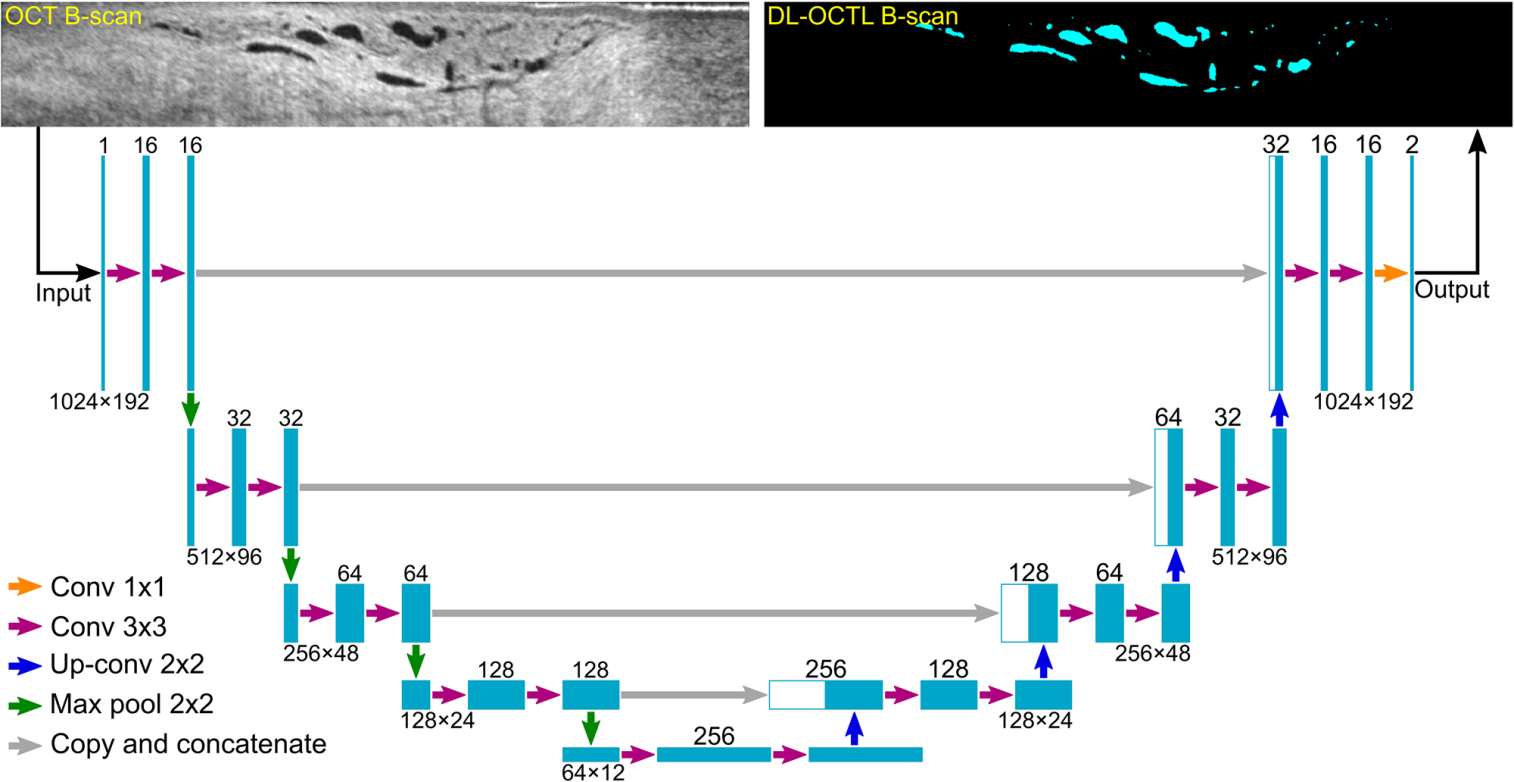
DL-OCTL structure based on U-Net. The left and right half of the network are corresponding to the contracting path and expansive path, respectively. The width of the cyan blocks indicates the numbers of the feature channels as specified on the top. The height of the cyan blocks indicates the dimension of the images as specified below the blocks as width × height in pixel number.

The DL-OCTL method was developed based on the U-Net network originally proposed by Ronneberger et al.^26^. This network has a symmetric structure comprising two major parts as shown in the left and right streams in Fig. 2, including the contracting path and the expansive path, respectively. In the contracting path, the original OCT B-scan data (single-precision floating-point numbers) were first converted to 8-bit unsigned integers (i.e., grayscale image data) as the direct input and then convolved with two 3 × 3 kernels (purple arrows) to extract the low-level semantic information. Subsequently, a 2 × 2 max pooling operation (green arrow) was performed, which was followed by another two 3 × 3 convolutions. This formed a downsampling process to halve the feature map in width and height dimensions and to double the number of feature channels. A total of 4 downsampling processes were performed with respectively 32, 64, 128 and 256 feature channels generated from each instance of downsampling, as shown in Fig. 2.

Symmetrically, the expansive path (right stream in Fig. 2) restored the size of the images by upsampling for 4 times, each of which comprised a 2 × 2 up-convolution (blue arrows) to double the feature map in width and height dimensions and to halve the number of the feature channels, and two subsequent 3 × 3 convolutions (purple arrows). Skip connections (gray arrows) were introduced to the expansive path to fuse feature maps at corresponding positions in the two paths, in order to compensate for the information lost during downsampling in the contracting path. This provided higher resolution information during upsampling in the expansive path and improved the segmentation accuracy. A 1 × 1 convolution was used in the final layer to achieve the desired number of classes (i.e., 2 classes including vessel pixel and non-vessel pixel), in which a single pixel can only be either a vessel pixel or non-vessel pixel. The structure of the network shown in Fig. 2 allowed low-resolution feature images to be mapped into the pixel-level segmentation results.

Among the 23 acquired 3-D OCT scans (19,736 B-scans) from the 11 eye samples, one 3-D OCT scan comprising 600 B-scans was chosen to provide high OCT image quality with minimal OCT artifacts (e.g., due to the tissue surface reflection) and accurately segmented vessels (i.e., vessel labels) by conventional OCTL. This 3-D OCT scan comprised dominant lymphatic vessels and a small number of aqueous vein vessels, which had similarly low OCT signal and barely distinguishable vessel cross-sections between the two vessel types in the OCT B-scans. Among the 600 B-scans in this 3-D scan, 300, 150 and 150 B-scans were randomly allocated for the training, validation and initial testing of the DL-OCTL method, respectively. Augmentation was not used during training as the target regions were small and scattered in the large background region. Subsequently, testing was more comprehensively performed on the remaining 22 3-D OCT scans with 19,136 B-scans (testing group as shown in Fig. 1b which was not used for training) mostly from other eye samples to assess the performance across different tissue regions and eye samples, by a comparison to the conventional OCTL. The DL-OCTL method was implemented on a GPU (NVIDVIA Tesla P100 PCIe 16 GB, 2016 A1) using PyTorch. The training and validation results, a series of comparison cases from the testing group and an overall summary of DL-OCTL results are shown in the following “Results”.

### Assessment metrics

To quantify the performance of DL-OCTL as compared to conventional OCTL, Intersection over Union (IOU) was calculated between the corresponding DL-OCTL and conventional OCTL B-scans, indicating how well the segmented vessels by the two methods overlap with each other. In particular, the vessel label images from conventional OCTL were converted into tensors and compared to the corresponding tensors from DL-OCTL, to calculate IOU using PyTorch. The IOU was provided for the imaging cases and summarized for all the testing data used in this study in the “Results” section. In addition, the mean IOU was calculated among the B-scans in the training and validation groups as the accuracy, respectively, while their loss was calculated using the standard Dice Loss.

As vessel density is a commonly used parameter in vessel imaging especially by OCT angiography (OCTA) imaging of blood vessels, we also measured the vessel area density in the 2-D projection images and volume density in the 3-D DL-OCTL and OCTL volumes. The density was calculated as the ratio of the total vessel area/volume to the total tissue area/volume. It should be noted that the area and volume density were mainly used to indicate the abundance of the vessels for the imaging cases shown in “Results”, which could not assess the overlap of the segmented vessels by the two methods as IOU.

## Results

### Training and validation of DL-OCTL

**Figure 3:**
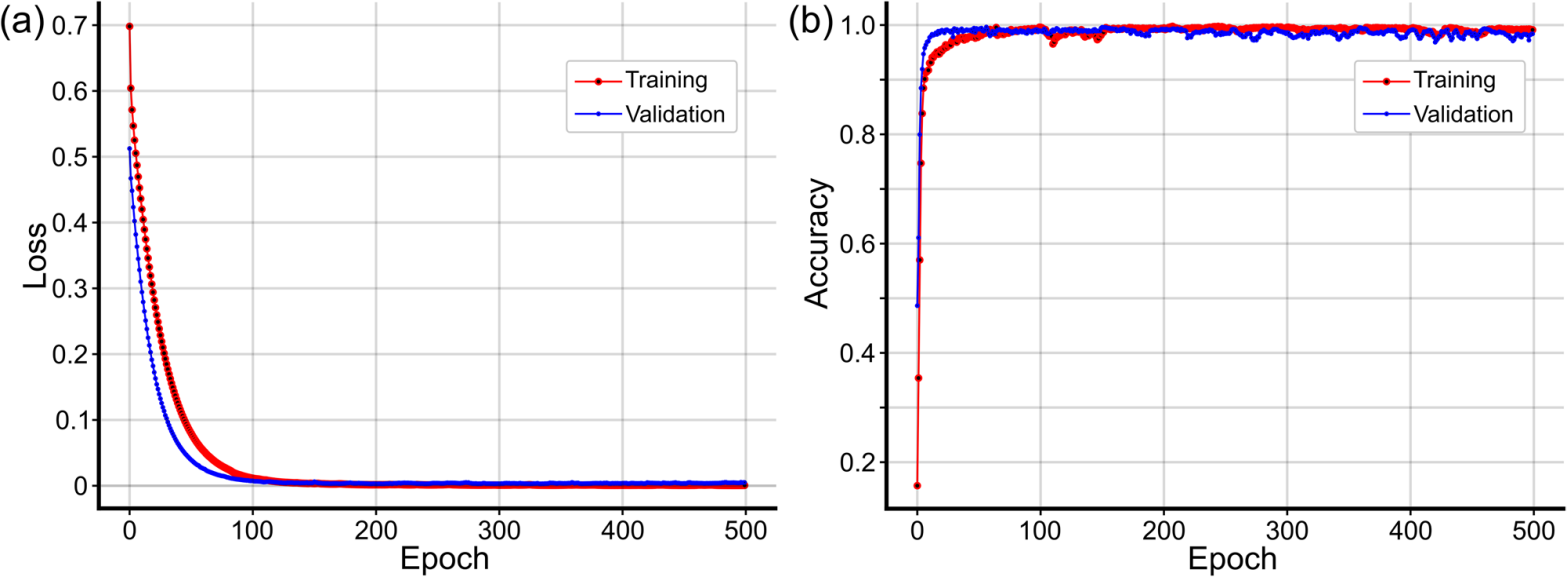
The loss curve (**a**) and accuracy curve (**b**) of the DL-OCTL model for the training (in red) and validation (in blue).

In the training of the DL-OCTL method using OCT B-scans from the training group, the Adam optimizer was used to update the parameters of the DL-OCTL model^31^, where the initial learning rate was set to 0.0001. Figure 3a,b show the loss and the accuracy curves of the DL-OCTL model during training and validation. As shown in Fig. 3, the DL-OCTL model has a strong learning ability (i.e., loss approaching zero and accuracy approaching one with good stability after approximately 100 epochs). Although the number of B-scan images used in the training (*n* = 300) is not large, the model still has a good fitting capability and does not overfit (i.e., validation loss does not increase after it first approaches zero). Therefore, cross-validation was not used in this study. When the model has been trained for approximately 100 epochs, the loss and the accuracy curves of the model have already converged to the best states, demonstrating the good performance of the DL-OCTL method.

### Imaging of lymphatic vessels

**Figure 4:**
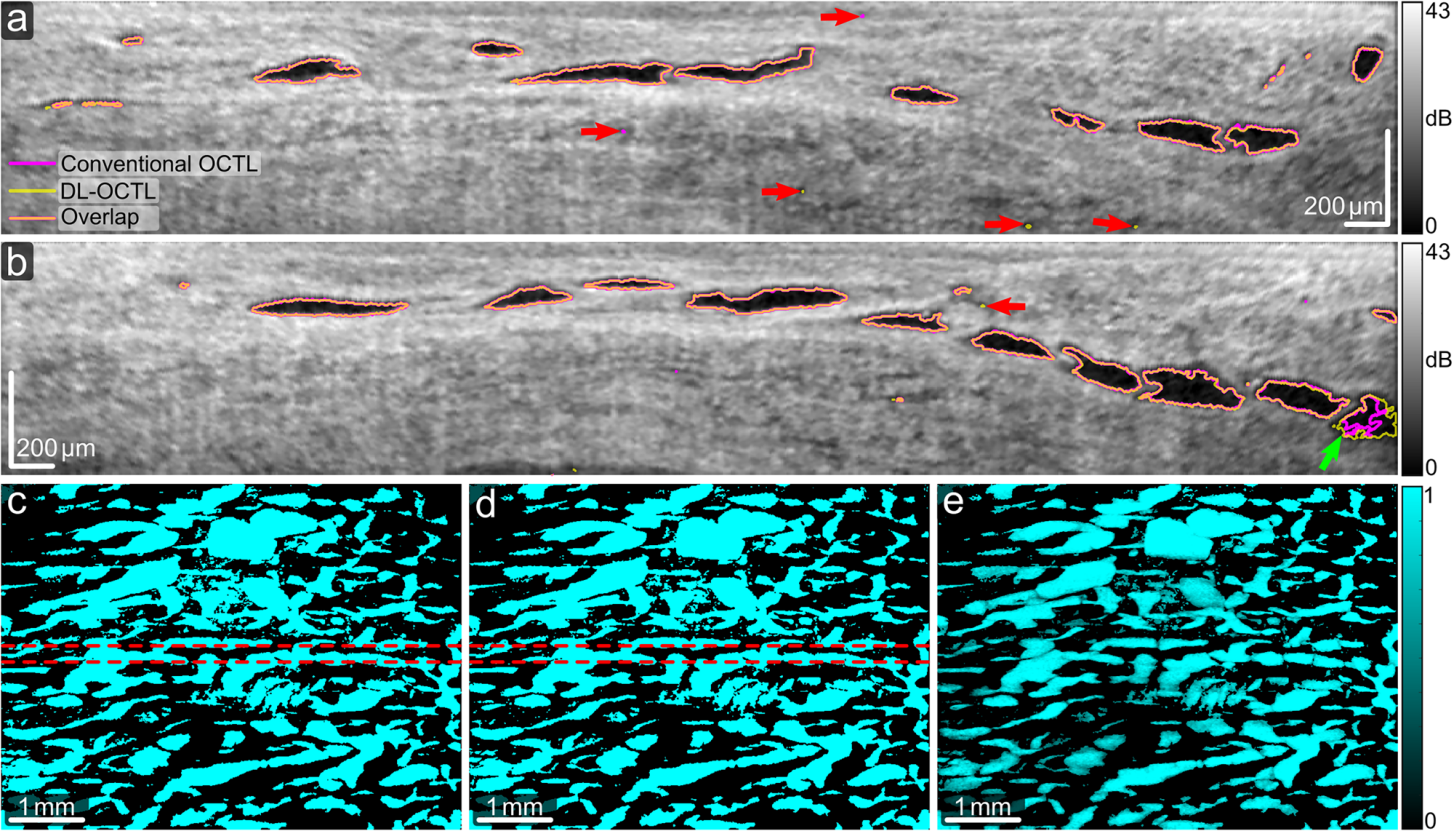
OCTL imaging of a region in the porcine eye with dominant lymphatic vessels. (**a**,**b**) OCT B-scans with the outlines of the segmented vessel cross-sections marked in magenta and yellow for conventional OCTL and DL-OCTL, respectively. Orange outlines mark the overlapped segmentations by the two OCTL methods. Red and green arrows mark regions with different segmentations by the two methods. (**c**–**e**) Projection of vessels from the tissue surface to a depth of 300 µm obtained with conventional OCTL, DL-OCTL and DL-OCTL after weighting, respectively. The top and bottom red dashed lines in (**c**) and (**d**) indicate the locations of the B-scans in (**a**) and (**b**), respectively.

The scanned tissue regions of the eye samples can comprise mainly lymphatic vessels, mainly aqueous vein vessels, or a mixture of both vessels in a single OCT scan. The morphological differences between these two vessel types were reported in a previous study^16^. Figure 4 shows OCTL imaging of a tissue region with mainly lymphatic vessels in one eye sample, characterized by irregular vessel shapes and uneven diameters along the vessel segments in the projection images of the vessels^16^. The B-scans in Fig. 4a,b show the segmentation results from DL-OCTL, as compared to those from conventional OCTL. The outlines of the segmented vessel cross-sections in the B-scans are marked in magenta for conventional OCTL and in yellow for DL-OCTL. The transparency and width of the yellow outlines are adjusted so that an orange color is observed when they overlap with the magenta outlines, indicating high similarity between the two methods.

In Fig. 4a,b, the two OCTL methods present very similar vessel segmentations (i.e., orange outlines) with minor differences at small local regions with low OCT signal due to non-vessel structures, such as those marked by the red arrows. DL-OCTL also shows improved segmentation of one big vessel as marked by the green arrow in Fig. 4b. The averaged Intersection over Union (IOU) of the segmentation by the two methods in all 600 B-scans within the OCT volume is 0.77, indicting an overlap of most segmented vessel cross-sections and similar performance of the two methods.

The en face projection images of the segmented vessels in the binary OCTL volumes are shown in Fig. 4c,d from conventional OCTL and DL-OCTL, respectively. The two images show consistent vessel structures with very minor variations as small regions of noise, leading to a similar vessel area density (41.6% for DL-OCTL vs 42.0% for conventional OCTL). In the OCTL volumes, the calculated vessel volume density from the tissue surface to a depth of 300 µm is also consistent between DL-OCTL (3.6%) and conventional OCTL (3.7%), demonstrating the very comparable performance of DL-OCTL to conventional OCTL. To improve the visualization of the vessel connections in the projection images, the binary DL-OCTL volume was further weighted by the reversed signal intensity of the corresponding OCT scan (i.e., vessel regions with an originally low OCT signal show high reversed OCT signal intensities for weighting), and subsequently used for projection. The resulting en face projection image is shown in Fig. 4e and is referred to as weighted projection hereinafter.

**Figure 5:**
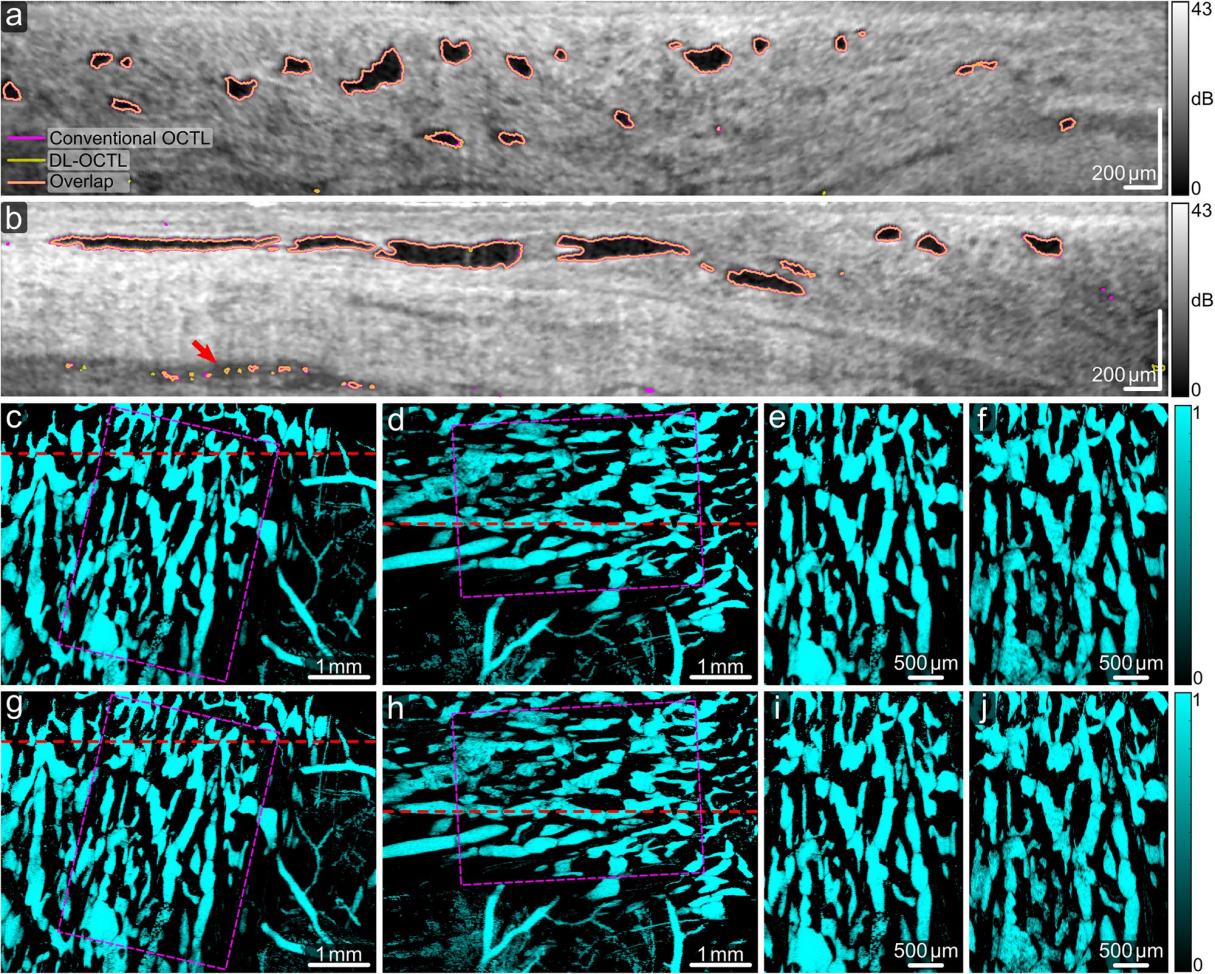
OCTL imaging of a region in the porcine eye with dominant lymphatic vessels by different scanning orientations. (**a**,**b**) OCT B-scans with the outlines of the segmented vessel cross-sections marked in magenta and yellow for conventional OCTL and DL-OCTL, respectively. Orange outlines mark the overlapped segmentations by the two OCTL methods. The red arrow marks a region with segmentation artifacts by the two methods. (**c**,**d**) Weighted projection of vessels from the tissue surface to a depth of 300 µm obtained with DL-OCTL from the perpendicular and parallel orientations of the B-scans relative to the main axis of the vessels, respectively. The red dashed lines in (**c**) and (**d**) indicate the locations of the B-scans in (**a**) and (**b**), respectively. (**e**,**f**) Registered weighted projection images from (**c**) and (**d**) as marked by the magenta outlines, respectively. (**g**–**j**) Weighted projection images obtained with conventional OCTL corresponding to (**c**–**f**), respectively.

The segmentation of the vessels by DL-OCTL is performed based on B-scans, which could comprise distinct shapes of vessel cross-sections, depending on the B-scan orientation relative to the main axis of the vessel segments. To assess if the performance of DL-OCTL is impacted by the shapes of the vessel cross-sections and B-scan orientation, another eye tissue region was scanned with two different B-scan orientations: one orientation perpendicular and the other parallel to the main axis of the vessel segments. As shown in Fig. 5a, the perpendicular orientation leads to different shapes of the vessel cross-sections from the long thin structures in Fig. 5b in the parallel orientation. In both orientations, DL-OCTL showed largely consistent results with conventional OCTL, although several local artifacts pop up, both in conventional OCTL and DL-OCTL, as indicated by the red arrow in Fig. 5b. The consistency indicates that DL-OCTL’s performance is not impacted by the shapes of the vessel cross-sections and thus not sensitive to the B-scan orientation. This is further validated by the weighted projections of the vessel networks by DL-OCTL in Fig. 5c,d, and the registered images in Fig. 5e,f from the perpendicular and parallel orientations, respectively, via rigid translation and rotation.

Figure 5g–j show the weighted projection images obtained using conventional OCTL, which are corresponding to Fig. 5c–f from DL-OCTL. These images indicate the very comparable imaging performance of DL-OCTL to the conventional OCTL method. In addition, conventional OCTL is not impacted by the B-scan orientation as well, which is expected as conventional OCTL is based on processing of individual A-scans, not B-scans as in DL-OCTL.

### Imaging of aqueous veins

**Figure 6:**
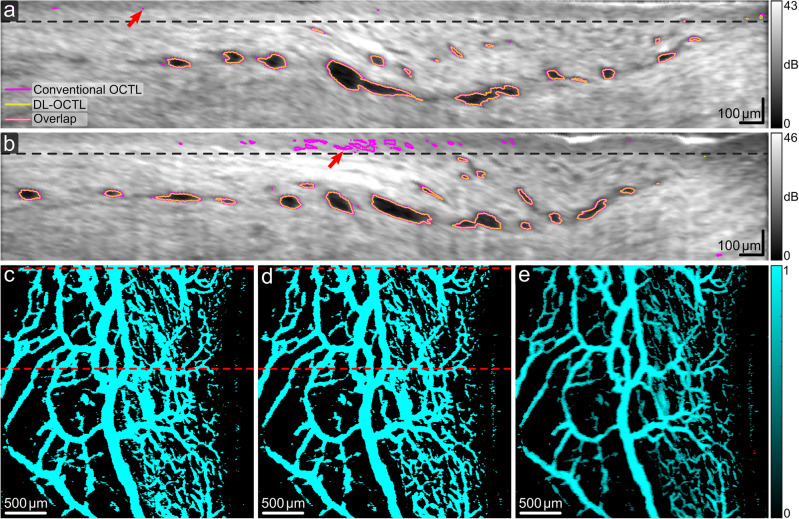
OCTL imaging of a region in the porcine eye with dominant large aqueous vein vessels. (**a**,**b**) OCT B-scans with the outlines of the segmented vessel cross-sections marked in magenta and yellow for conventional OCTL and DL-OCTL, respectively. Orange outlines mark the overlapped segmentations by the two OCTL methods. Red arrows mark regions with segmentation artifacts by conventional OCTL in the superficial region up to a depth of 75 µm (black dashed lines). (**c**–**e**) Projection of vessels from a depth of 75 µm to 400 µm obtained with conventional OCTL, DL-OCTL and DL-OCTL after weighting, respectively. The top and bottom red dashed lines in (**c**) and (**d**) indicate the locations of the B-scans in (**a**) and (**b**), respectively.

**Figure 7:**
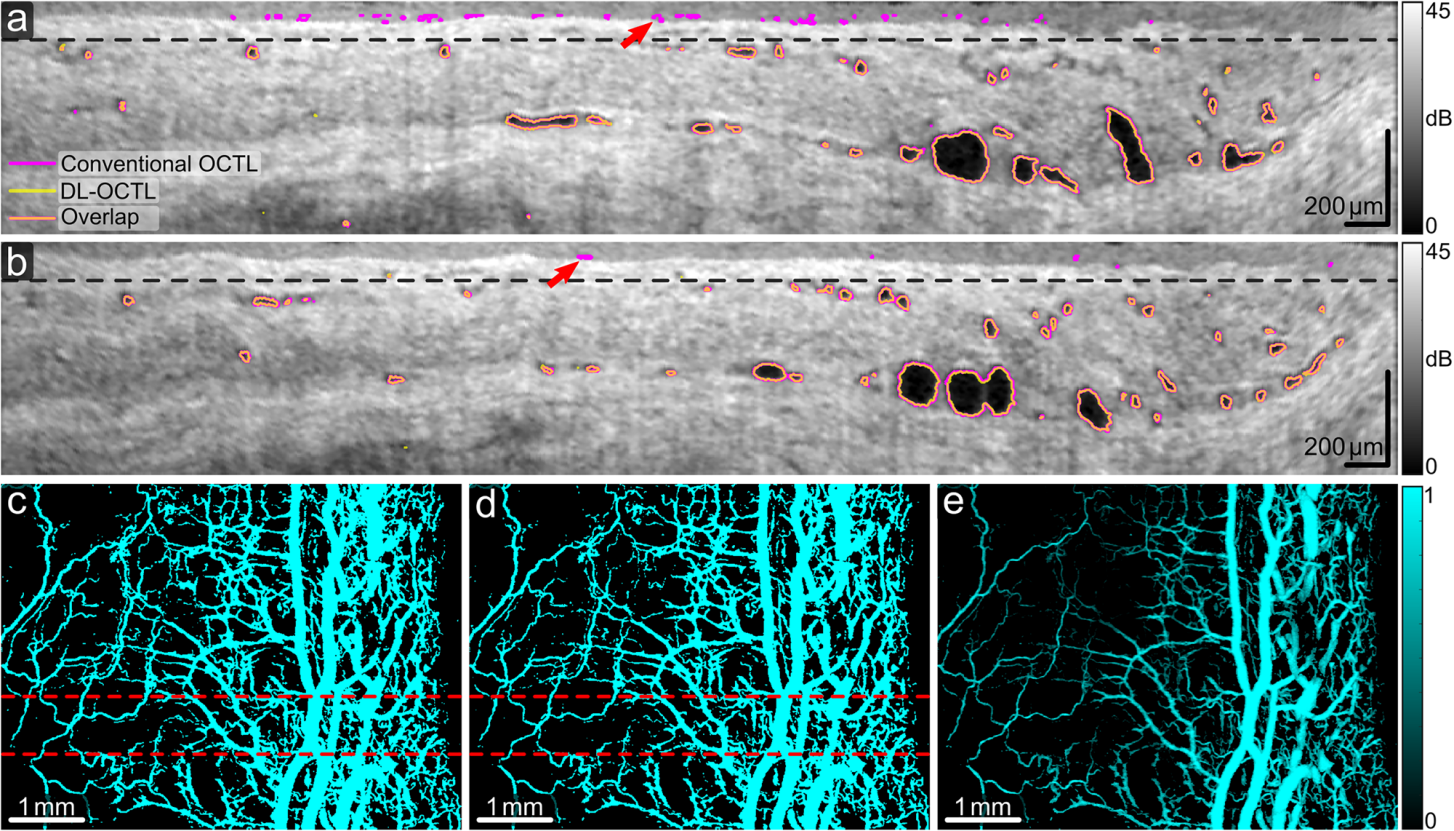
OCTL imaging of a region in the porcine eye with both large and small aqueous vein vessels. (**a**,**b**) OCT B-scans with the outlines of the segmented vessel cross-sections marked in magenta and yellow for conventional OCTL and DL-OCTL, respectively. Orange outlines mark the overlapped segmentations by the two OCTL methods. Red arrows mark regions with segmentation artifacts by conventional OCTL in the superficial region up to a depth of 75 µm (black dashed lines). (**c**–**e**) Projection of vessels from a depth of 75 µm to 400 µm obtained with conventional OCTL, DL-OCTL and DL-OCTL after weighting, respectively. The top and bottom red dashed lines in (**c**) and (**d**) indicate the locations of the B-scans in (**a**) and (**b**), respectively.

Two imaging cases comprising mainly aqueous veins are shown in Fig. 6 with mostly large vessels and in Fig. 7 with a mixture of both large and small vessels. OCTL imaging of the first case in Fig. 6 shows regular vessel shapes and diameters along the vessel segments in the projection images^16^. While most of the vessels are detected with both DL-OCTL and conventional OCTL in the B-scans in Fig. 6a,b, differences are also observed in the top thin epithelium layer that presents a lower OCT signal than the underlying tissue, such as those marked by the red arrows. These differences are corresponding to segmentation artifacts introduced by conventional OCTL, when the tissue presents significant heterogeneity along the depth, invalidating the single-scattering model used in attenuation compensation. As only the vessel labels without such artifacts were used for the training of the DL-OCTL method, these artifacts are largely eliminated by DL-OCTL for a more accurate segmentation in Fig. 6a,b. As a consequence, the IOU of the segmentation by the two OCTL methods among all B-scans in the 3-D OCT scan is reduced to 0.70.

The projection image of the segmented vessels by DL-OCTL in Fig. 6d is similar to that obtained by conventional OCTL in Fig. 6c, when the projection depth (from 75 to 400 µm below the tissue surface) was selected to intentionally eliminate the depth range containing the artifacts in conventional OCTL. The resulting vessel area density in the projection image from DL-OCTL (32.8%) agrees well with that from the conventional OCTL (34.5%). The volume densities of the segmented vessels in the 3-D OCTL scans are also similar (2.4% and 2.6% for DL-OCTL and conventional OCTL, respectively) for the depth range without the segmentation artifacts in conventional OCTL. These comparisons show that DL-OCTL achieves a very comparable performance to the conventional OCTL for imaging aqueous vein vessels, while mitigating the segmentation artifacts in conventional OCTL.

Figure 7 shows OCTL imaging of a tissue region with dominant aqueous vein vessels in another eye sample. Despite the much smaller vessel dimensions and the reduced vessel contrast in the B-scans in Fig. 7a,b as compared to the case in Fig. 6, DL-OCTL still presents a consistent segmentation with conventional OCTL. Similar to the case in Fig. 6, the segmentation artifacts in the superficial epithelium (marked by red arrows) by conventional OCTL are eliminated by DL-OCTL. The projection images in Fig. 7c,d show very similar vessel networks by the two OCTL methods and similar vessel area densities (32.7% and 33.8% for DL-OCTL and conventional OCTL, respectively), when the tissue depth range comprising imaging artifacts by conventional OCTL was excluded. The weighted projection from DL-OCTL in Fig. 7e presents a clear visualization of the vessel connections inside the network, although it seems to show less visible vessels than those in Fig. 7d due to the increased OCT signal intensity inside the segmented small vessels. A close examination indicates that the vessels are largely maintained after weighting, although with a reduced vessel signal intensity in Fig. 7e.

### Imaging summaries

Consistent with the four cases shown above, DL-OCTL imaging, applied to 3-D scans from 11 eye samples in this study, overall shows very comparable imaging performance to the conventional OCTL in both the B-scans and projection images. The IOU of the segmentation by the two OCTL methods across all B-scans within each 3-D scan was calculated, leading to a value of 0.79 ± 0.071 (mean ± standard deviation). This further indicates the good imaging performance of DL-OCTL, as compared to conventional OCTL. The average processing time by DL-OCTL is approximately in the range of 26–35 ms for one B-scan. This is significantly faster than the conventional OCTL by thresholding with attenuation compensation which requires ~ 350 ms on average to process one B-scan. It is difficult to give a fair comparison of the processing time due to the different implementations (i.e., CPU for conventional OCTL and GPU for DL-OCTL). However, these figures could still provide a rough comparison as both implementations used moderate computing power in their own types.

## Discussion

This study presents the first adaptation of U-Net into a DL-OCTL method for direct segmentation of the lymphatic and aqueous vein vessels in OCT scans of the eye tissue. The DL-OCTL method demonstrated very comparable performance to the conventional OCTL method based on thresholding with attenuation compensation. In comparison, DL-OCTL eliminates the segmentation artifacts in conventional OCTL which are associated with tissue heterogeneity. In conventional OCTL, the OCT signal modelling used in attenuation compensation assumes that the tissue is homogenous along the depth direction. When this assumption becomes invalid, the tissue heterogeneity can lead to invalid modelling of the OCT signal and the resulting artifacts. These artifacts are prominent in the superficial regions in Figs. 6b and 7a, which are largely eliminated by DL-OCTL. This improvement is consistently observed in B-scans with such artifacts caused by conventional OCTL in this study, although this improvement was not quantified. In addition, DL-OCTL improves the processing speed and does not quire OCT-related knowledge for correct implementation as in conventional OCTL. With these favorable features, DL-OCTL promises to provide improved practicality for future pre-clinical and clinical label-free imaging of lymphatic and aqueous vein vessels.

DL-OCTL provided clear visualization of the lymphatic vessels and aqueous vein vessels in the eye, both of which are associated with glaucoma but with potentially different applications. The aqueous veins in the sclera form an important part of the outflow pathway of the AH to regulate the IOP^21^. DL-OCTL imaging of the aqueous veins may provide new insights into the pathogenesis and potential therapeutic strategy of glaucoma, in which elevated IOP is a major risk factor. The lymphatics in the conjunctiva of the eye are considered critical for the longevity of the created drainage pathway by filtration surgery for glaucoma^3^. DL-OCTL’s capability of imaging the lymphatics may help to understand the roles of lymphatics in filtration surgery and may lead to improved treatment outcomes by guiding the selection of the optimal region with lymphatics for surgery. While the ex vivo imaging in this study demonstrated the potential of DL-OCTL, further development is required to translate it to in vivo imaging of human eyes, based on faster scanning to mitigate tissue motion artifacts^32,33^.

OCTL, including DL-OCTL, provides a distinct imaging function from the more common OCTA. OCTA uses the temporal changes of the OCT signal induced by the blood flow to identify blood vessels, which have moderate to high OCT signal caused by the rich scatterers. However, OCTA is not effective for imaging the lymphatic vessels and aqueous veins due to the lack of scatterers in the lymph and aqueous humor (i.e., extremely low OCT signal). OCTL instead uses the characteristic low OCT signal to segment the lymphatics and aqueous veins. Although the imaging capability was demonstrated ex vivo, the vessel contrast mechanism of OCTL is expected to be feasible for in vivo imaging as the circulating lymph and aqueous humor do not have rich scatterers in vivo. In addition, OCTA requires the acquisition of two or more repeated OCT B-scans from the same tissue location, which can be easily affected by tissue motion. In comparison, OCTL may be less impacted by motion when it is translated to in vivo imaging as it only requires one B-scan from each tissue location. Apart from fast scanning techniques, the motion reduction methods previously developed for OCT/OCTA imaging can also be adopted to mitigate motion for in vivo OCTL imaging^34–36^.

Whilst there are multiple U-Net architectures available, the selected architecture in this study aimed to maximize the practicality of the DL-OCTL method for future clinical use. In such scenarios, DL-OCTL will be integrated into the OCT system with limited computing power and the memory. Thus, the DL-OCTL method will need to be efficient for limited computing power with a low memory usage, and still provide a fast-imaging capability. The architecture was selected to best meet these criteria. Although other alternative architectures may provide an improved image quality, the reduced practicality may preclude the translation to clinical use.

One limitation of DL-OCTL and conventional OCTL for eye imaging is the lack of automatic differentiation of the lymphatic and aqueous vein vessels. Both lymph and aqueous humor present similarly high optical transparency and thus indistinguishably low OCT signal. However, the distinct morphological features of the vessels could aid the differentiation of the two vessel types: lymphatic vessels show largely irregular vessel shapes, uneven diameters along the vessel segments and local discontinuity due to valves, as compared to the aqueous vein vessels^16^. These features form the basis for the current manual differentiation of the two vessel types. Future work could seek to use deep learning to automatically differentiate these two vessels. Another limitation is that tissue perfusion was not used during OCT scanning^20^, which might lead to nonoptimal vessel contrast. With care taken to avoid pressure on the tissue and to complete the scanning while the samples were fresh (within ~ 3–4 h of enucleation), the vessels largely showed good contrast for imaging as compared to previous studies^16,20^. The potential improvement of vessel contrast by perfusion could be assessed in future work.

IOU was used as one assessment of how well the segmentation by DL-OCTL agreed with that by conventional OCTL. A value of 0.79 ± 0.071 was achieved across all scans, indicating overall a high degree of overlap of the segmented vessels by the two methods and thus similar imaging performance. One major contributor to the non-overlapping (i.e., different) segmentations between the two methods was the imaging artifacts in conventional OCTL as shown in the “Results”. These artifacts indicate one limitation of conventional OCTL based on thresholding with attenuation compensation that the assumption of the tissue homogeneity along depth in attenuation compensation can become invalid. The DL-OCTL method was designed to mitigate such imaging artifacts by using vessel label images free of such artifacts for the training. Although this approach reduced the IOU, a more accurate segmentation was achieved by DL-OCTL as an advantage.

Projection images of the vessels were taken to demonstrate DL-OCTL’s capability to visualize the vessel network and to quantify the vessel density, as compared to conventional OCTL. The projection images visually showed a higher consistency of the two methods than that indicated by the IOU calculated in the B-scans. First, this was due to the exclusion of the depth range with the segmentation artifacts in the superficial tissue by conventional OCTL from the depth range for projection. Second, projection tended to mitigate the differences of the segmented vessels along the depth direction. Yet, projection is widely used in similar imaging techniques, such as in OCTA^37,38^ and may still be a useful visualization tool for future clinical applications of DL-OCTL.

Validation of lymphangiography for imaging the micro-scale lymphatics is still challenging due to the lack of an imaging technique to provide the ground truth. In this study, conventional OCTL based on thresholding with attenuation compensation was used as the best available ‘ground truth’ for the development and validation of DL-OCTL, as conventional OCTL previously showed promising imaging performance^16^. The DL-OCTL method demonstrated comparable results to conventional OCTL with reduced imaging artifacts that were caused by inaccurate OCT signal modelling associated with tissue heterogeneity. However, as the ‘ground truth’ is not perfect and comprises segmentation artifacts, both conventional OCTL and DL-OCTL methods might miss vessel segments that present poor vessel contrast (i.e., reduced optical transparency) and/or have very small dimensions. Future work can seek to improve the accuracy of the vessel labels by incorporating manual segmentation so as to develop a more accurate DL-OCTL method.

## Conclusion

In conclusion, we demonstrated a DL-OCTL method for label-free imaging of the lymphatic and aqueous vein vessels in the eye with OCT. The DL-OCTL method, developed based on the U-Net network, showed promising imaging performance on ex vivo eyes, as compared to the previously proposed conventional OCTL method based on thresholding with attenuation compensation. In contrast to conventional OCTL, DL-OCTL mitigated the imaging artifacts that were caused by inaccurate OCT signal modelling associated with tissue heterogeneity, accelerated the processing speed and eliminated the requirement for OCT-related knowledge of the user for correct implementation. These improvements could collectively enhance the practicality of OCTL, potentially providing a more viable tool for preclinical and clinical applications.

## Data Availability

The datasets generated and analyzed in this study are not publicly available at this time but may be obtained from the corresponding authors upon reasonable request.
